# Growth Factor Dependent Regulation of Centrosome Function and Genomic Instability by HuR

**DOI:** 10.3390/biom5010263

**Published:** 2015-03-20

**Authors:** Natalia Filippova, Xiuhua Yang, Louis Burt Nabors

**Affiliations:** Department of Neurology, University of Alabama at Birmingham, 510 20th Street South, FOT 1020, Birmingham, AL 35209, USA; E-Mails: as1999@uab.edu (N.F.); xiuhua@uab.edu (X.Y.)

**Keywords:** ABL tyrosine kinase, cell signaling, centrosome, genomic instability, RNA binding protein, Src

## Abstract

The mRNA binding protein HuR is over expressed in cancer cells and contributes to disease progression through post-transcriptional regulation of mRNA. The regulation of HuR and how this relates to glioma is the focus of this report. SRC and c-Abl kinases regulate HuR sub-cellular trafficking and influence accumulation in the pericentriolar matrix (PCM) via a growth factor dependent signaling mechanism. Growth factor stimulation of glioma cell lines results in the associate of HuR with the PCM and amplification of centrosome number. This process is regulated by tyrosine phosphorylation of HuR and is abolished by mutating tyrosine residues. HuR is overexpressed in tumor samples from patients with glioblastoma and associated with a reduced survival. These findings suggest HuR plays a significant role in centrosome amplification and genomic instability, which contributes to a worse disease outcome.

## 1. Introduction

The centrosome is a small intracellular organelle, which duplicates once in each mitotic cycle to form the microtubule-organizing centers (MTOC) that are responsible for the segregation of chromosomes during mitosis and cytokinesis [1]. In non-proliferating cells, the centrosome regulates cytoskeletal dynamics and cell migration [2,3]. Disruption of centrosome function and regulation is the cause of several neurological diseases, congenital and developmental, as well as cancer [4,5,6,7]. Several well established families of oncogenes (growth factors: EGF, bFGF, PDGF; growth factor receptors: ErbB/HER, PDGFR; tyrosine kinases: SRC, Yes1, Lyn, ILK, Fyn, EGFR; mitotic check points molecules: p53, NDC80, PTEN; and serine/threonine protein kinases AURKA, PLK1, NEK2) provide molecular links between centrosome abnormalities and the development of cancer [8,9,10,11,12,13,14,15].

In our recent report, we identified the mRNA binding protein HuR as a new regulator of the centrosome in cancer cells [16]. HuR is over-expressed in tumor cells and is essential for mRNA splicing, stabilization, and translation [17,18,19,20,21,22]. In glioma cell lines, the HuR protein co-localizes with the PCM and controls mRNA stabilization and translation in proximity to the centrosome [16]. An analysis of the database of centrosomal proteins and potential HuR mRNA targets reveals 76 genes that contain binding sites for HuR on 3' UTR and are permanently or transiently co-localized with the PCM [23,24]. These genes are regulators of centrosome amplification, clustering (phenomenon which is specific for cancer cells), and centrosomes/chromosomes integrity during mitosis [7,9,25].

The initiation of centrosome duplication requires the activation by growth factor signaling pathways in mitotic cells and is aberrant in cancer cells [11,12,13,14,26]. In the current manuscript, we evaluated the hypothesis that HuR may play a significant role in growth factor dependent regulation of centrosome amplification in cancer cells. HuR is phosphorylated at tyrosine residues in tumor cells [27,28,29] and has several consensuses for phosphorylation by Abl-1, SRC, Lyn, FAK, and EGFR tyrosine kinases, involved in aberrant regulation of centrosomes in cancer cells [8,15,30,31,32,33]. By using molecular and cellular approaches, we discovered a positive correlation between association of HuR with centrosomes and centrosome amplification induced by growth factors in U251 cells. We provide evidence that the association of HuR with PCM is a growth factor dependent process and is enhanced by HuR phosphorylation at tyrosine residues. We confirmed localization of Abl-1 and SRC tyrosine kinases in the PCM and direct interaction with HuR.

The centrosomal abnormalities resulting from mutations of check points molecules are the main cause of genomic instability in cancer cells which promotes tumor progression, unsuccessful treatment outcomes, and patient mortality [6,9,11,12,30]. The rate of genomic instability associated with gliobastoma (GBM) is high and positively correlates with hyperactivity of growth factor dependent pathways [34,35,36]. We found that the aberrant growth factor dependent HuR regulation in U251 cells is associated with the enhancement of centrosome amplification and an increase in the rate of genomic instability. We confirmed a significant decline of growth factor dependent centrosome amplification and micronuclei formation following decrease of HuR expression. By using site directed mutagenesis and partial substitution of endogenous HuR protein to a modified HuR-Y/5,95,105,200/F, we demonstrate a reduction in genomic instability and confirmed the loss of growth factor dependent HuR-Y/5,95,105,200/F regulation in cytoskeleton fraction.

In summary, we discovered a novel mechanism underlying the abnormal centrosome amplification and genomic instability in cancer cells.

## 2. Results and Discussion

### 2.1. Centrosome Amplification Evoked by Growth Factor Stimulation

**Figure 1:**
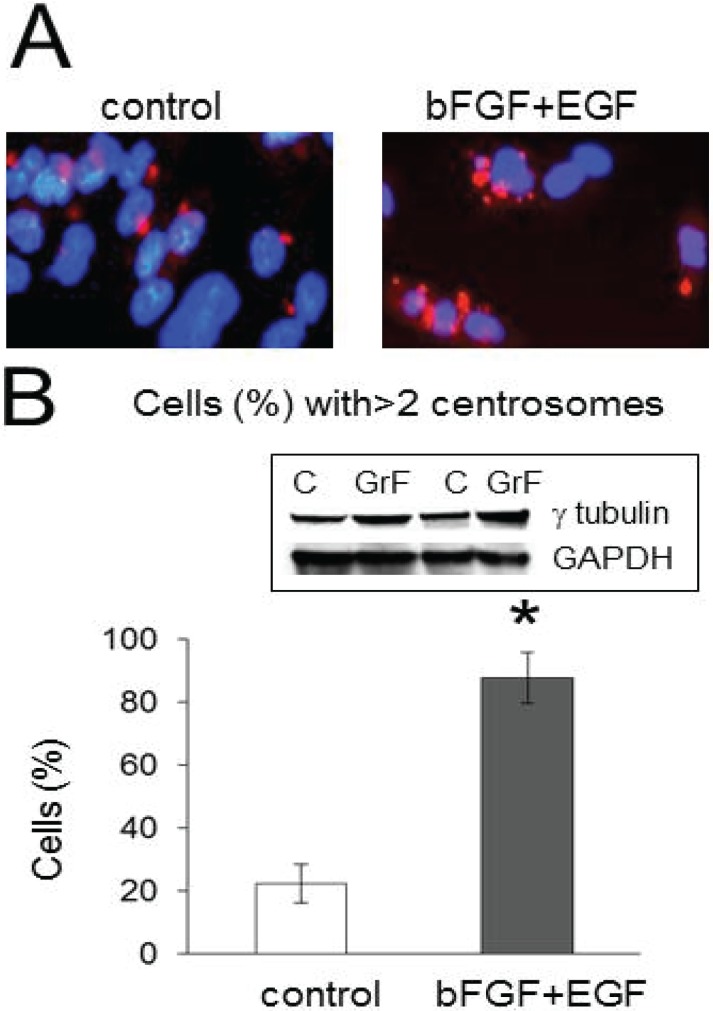
Stimulation with growth factors induces centrosome amplification in U251 cells. (**A**) Examples of centrosomes marked with the PACT domain of pericentrin attached to the red fluorescence protein (PACT-mKO1) in clones of U251 cells in control (unstimulated) or stimulated with growth factors (EGF, 40 nM and bFGF 40 nM). There is amplification of centrosomes in cells treated with growth factors compared to the control. Nuclei were stained with DAPI. (**B**) The graph represents the averaged percentages of cells with amplified centrosome number following stimulation with growth factors (87% ± 8%) *versus* control (22% ± 6%). The difference is significant with *p* = 0.0008. The experiments were performed four times. The insert illustrates the total level of γ-tubulin values in cells non-treated (C) and treated with growth factors (GrF). Note that growth factor treatment induced an increase of γ-tubulin/GAPDH ratio (by 44% and 57% in two illustrated experiments in Western blot). The increase is significant (50% ± 7%, *p* = 0.005, three experiments).

The amplification of centrosomes is growth factor dependent and can be induced by EGF and bFGF stimulation of cancer cells [10,11,12,13,14]. Figure 1 illustrates the amplification of centrosomes marked with the PACT domain of pericentrin in clones of U251 cells treated with bFGF (40 nM) and EGF (40 nM) for 48 h. We observed that 87% ± 8% of U251 cells had more than two centrosomes per nucleus following stimulation with bFGF and EGF compared to 22% ± 6% of cells with more than two centrosomes per nucleus in the control condition. The difference is significant (*p* = 0.0008). Similar results have been achieved in four experiments.

### 2.2. Centrosomes Amplification Induced by Growth Factor Stimulation Is HuR-Dependent

**Figure 2:**
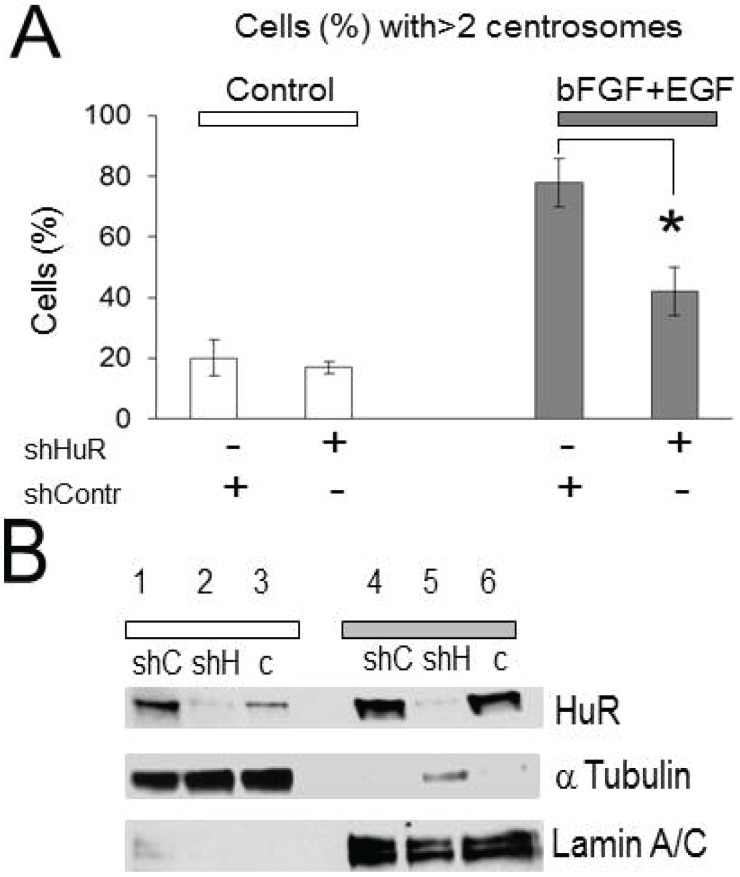
Knockdown of HuR diminishes centrosome amplification evoked by growth factors. (**A**) The graph represents the averaged percentages of cells with amplified centrosome number before and after HuR knockdown in the control condition and following stimulation with growth factors (20% ± 6% and 17% ± 2% (*n* = 3) *versus* 76% ± 8% and 42% ± 8% (*n* = 3)) in control and growth factors stimulated conditions, respectively. Note, that HuR knockdown significantly reduces the percentage of cells with amplified centrosome number in growth factor treated condition (*p* = 0.01), (**B**) Illustration of HuR knockdown in the cytoplasmic and nuclear fractions detected in Western blot. Lines 1 and 4 correspond to sh-control (shC), lines 2 and 5 correspond to sh-HuR (shH), and lines 3 and 6 correspond to non-transfected (**C**) conditions. The α-tubulin and lamin A/C mark cytoplasmic and nuclear fractions, respectively.

To determine whether HuR is involved in growth factor dependent centrosome amplification, the percentages of cells with amplified centrosome number were compared in the control condition (scrambled sh-control plasmid) *versus* HuR knockdown condition (shHuR plasmid). Figure 2A represents the averages of the percentages of cells with amplified centrosome number for each condition with and without treatment with growth factors. We did not observe significant alterations in centrosome amplification following HuR knockdown in cells not treated with growth factors (20% ± 6% and 17% ± 2% percent of cells in sh-control and sh-HuR conditions, respectively); however, the percentages of cells with amplified centrosome number significantly declined from 76% ± 8% to 42% ± 8%, *p* = 0.01 following HuR knockdown in cells treated with EGF and bFGF growth factors. The HuR knockdown was confirmed at the protein level by Western blot (Figure 2B). Note the significant decrease of HuR protein levels in both, nuclear and cytoplasmic fractions following HuR knockdown. Knockdown of the mRNA binding protein HuR attenuates centrosome amplification induced by growth factors.

### 2.3. HuR Association with Pericentriolar Matrix (PCM) Is Enhanced by Growth Factor Stimulation

**Figure 3:**
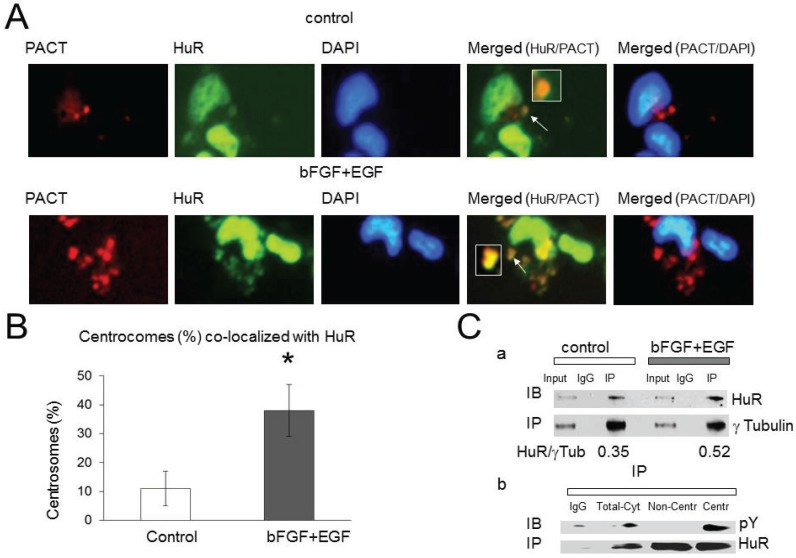
HuR association with the pericentriolar matrix (PCM) is enhanced by growth factors. (**A**) Examples of HuR-EGFP co-localization with centrosomes marked with the PACT domain of pericentrin attached to the red fluorescence protein (PACT-mKO1) in control and growth factor stimulated conditions. Nuclei were stained with DAPI. (**B**) The graph represents the averaged percentages of centrosomes co-localized with HuR-EGFP in control (11 ± 6, *n* = 4) and growth factor stimulated conditions (38 ± 9, *n* = 4). The difference is significant, *p* = 0.01; (**C**) (a) Illustration of HuR co-immunoprecipitation with γ-tubulin from cells non-stimulated and stimulated with growth factors. The IgG line illustrates the results of co-immunoprecipitation performed with rabbit IgG antibody as control. (**C**) (b) Illustration of HuR phosphorylation at tyrosine residues detected with pY antibody after HuR immunoprecipitation from cytoplasmic lysate (line Total-Cyt), non-centrosome fraction (line Non-Centr), and crude centrosome fraction (line Centr). Note the accumulation of pY-HuR protein in the crude centrosome fraction.

In our recent manuscript [16] we discovered HuR localization in the PCM and a HuR role in mRNA stabilization and *de-novo* protein synthesis in proximity to centrosomes. To determine if HuR co-localization with the PCM is growth factor dependent, we compared HuR (HuR-EGFP) co-localization with the PCM marked with PACT-mKO1 construct, in the presence and absence of growth factors using confocal scanning microscopy. Figure 3A illustrates examples of HuR-EGFP co-localization with PCM (PACT-mKO1) in both conditions. In the control condition, 11% ± 6% of analyzed centrosomes were co-localized with HuR; however, 38% ± 9% of centrosomes exhibited co-localization with HuR following EGF/bFGF treatment (Figure 3B). The enhancement of HuR co-localization with the PCM evoked by growth factors is significant (*p* = 0.01). At least 150 cells have been analyzed for each condition in each experiment.

In our next experiment, we immunoprecipitated the PCM by using γ-tubulin antibody from cells in control condition and following cell treatment with growth factors. We observed an increase in HuR co-immunoprecipitated with γ-tubulin following treatment with growth factors compared to the control condition (the HuR/γ-tubulin bands ratio was 0.52 in the presence of growth factors *versus* the HuR/γ-tubulin bands ratio 0.35 in non-treated cells, Figure 3Ca). Similar results have been observed in three experiments, the enhancement of HuR association with γ-tubulin in the presence of growth factors was significant (by 46% ± 20%, *n* = 3, *p* = 0.03) compared to non treated cells.

HuR immunoprecipitation from crude centrosomes fraction and from non-centrosome cytoplasmic fraction revealed a significant enhancement of HuR phosphorylation at tyrosine residues in the centrosome fraction compared to HuR phosphorylation in non-centrosome cytoplasmic fraction (the tyrosine dependent HuR phosphorylation has been detected by using p-Y antibody following HuR immunoprecipitation with HuR3A2 antibody) (Figure 3Cb). Similar results have been observed in two experiments.

These results demonstrate that EGF and bFGF stimulation promotes HuR phosphorylation at tyrosine residues and localization in the PCM.

### 2.4. Abl-1 and SRC Kinases Are Possible Regulators of HuR in the PCM

**Figure 4:**
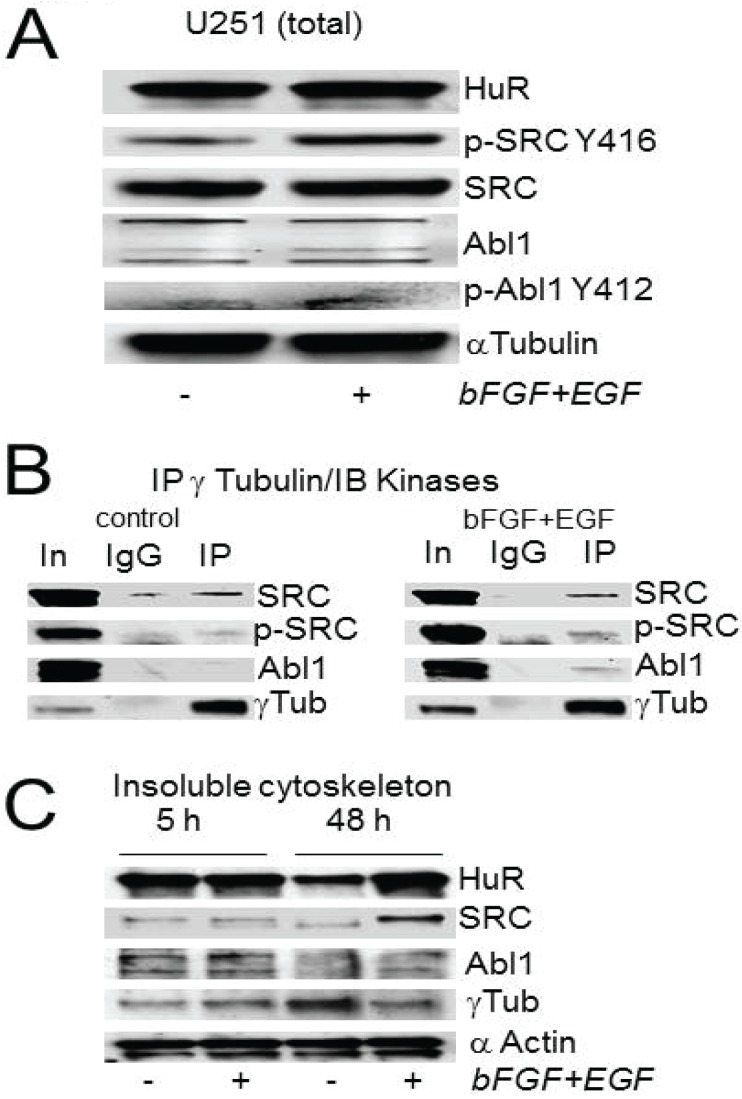
The association of Abl-1 and SRC kinases with the PCM and cytoskeleton is growth factor dependent. (**A**) Example of Western blot demonstrating the alteration of pY status in the catalytic domains of SRC and Abl-1 kinases induced by growth factor stimulation. (**B**) Co-immunoprecipitation of SRC and Abl-1 kinases with γ-tubulin in control and growth factor stimulated conditions. Note that the Abl-1 kinase exhibits significant enhance in co-immunoprecipitation with γ-tubulin following growth factor stimulation. (**C**) The contents of non-soluble cytoskeleton fractions obtained from control and growth factor stimulated (for 5 h and 48 h) conditions. There is a significant enhancement of HuR and SRC in the non-soluble cytoskeleton fraction following 48 h of cells treatment with growth factors compared to non-treated control.

To determine which tyrosine kinases may be involved in HuR regulation in PCM, we evaluated SRC, Abl-1 and EGFR families of tyrosine kinases (which may directly participate in growth factors dependent regulation of centrosomes [8,15,30,31,32,33]) in the contents of PCM co-immunoprecipitated with γ-tubulin antibodies from cells in the control condition and following growth factor stimulation. Figure 4A illustrates an increase in phosphorylation of key tyrosine residues in the catalytic domains of SRC and Abl-1 detected in total cell lysates of U251 cells after stimulation with EGF/bFGF compared to the non-treated condition. Note the significant increase in phosphorylation of SRC kinase at Y416 residue (on 67% ± 30%, *n* = 5, *p* = 0.011) and light increase of ABL1 kinase phosphorylation at Y412 residue (on 15% ± 14%, *n* = 4, *p* = 0.055) after cells treatment with growth factors. We did not observe significant alterations in total SRC and Abl-1 kinases protein levels following cell treatment with growth factors compared to non-treated cells (the values of SRC and c-Abl kinases normalized to the corresponding values in the non treated condition were 1.04 ± 0.09, *n* = 5 and 0.98 ± 0.04, *n* = 4, respectively).

Co-immunoprecipitation of PCM using γ-tubulin antibody revealed a significant enhancement in the association of Abl-1 kinase with the PCM following treatment with EGF/bFGF *versus* the control condition (Figure 4B). We did not observe a significant alteration in association of SRC kinase with γ-tubulin following treatment with growth factors (Figure 4B). However, the phosphorylation of Y416 residue of SRC kinase co-immunoprecipitated with γ-tubulin was increased (Figure 4B) and that may account for the alteration of SRC activity in the PCM following treatment with EGF/bFGF. Although we note a strong internalization of EGFR complexes after treatment with growth factors, we did not observe EGFR co-immunoprecipitation with γ-tubulin.

Figure 4C illustrates the appearance of SRC, Abl-1, HuR, and γ-tubulin in the insoluble cytoskeletal fractions in the control condition and following cell stimulation with growth factors for 5 h and 48 h. We observed a significant increase of HuR value (on 1.8 ± 0.5 folds, *p* = 0.05, three independent experiments) in the insoluble cytoskeleton fractions accompanied by an enhancement of SRC value (on 2.1 ± 0.6 folds, *p* = 0.03) following cell treatment with growth factors for 48 h compared to non-treated cells. This may reflect the facilitation of HuR and SRC subcellular trafficking induced by growth factors. Current data highlights Abl-1 and SRC kinases as possible HuR regulators in the PCM and in growth factor dependent sub-cellular trafficking.

### 2.5. Recombinant HuR Is Phosphorylated by Abl-1 and SRC Kinases

**Figure 5:**
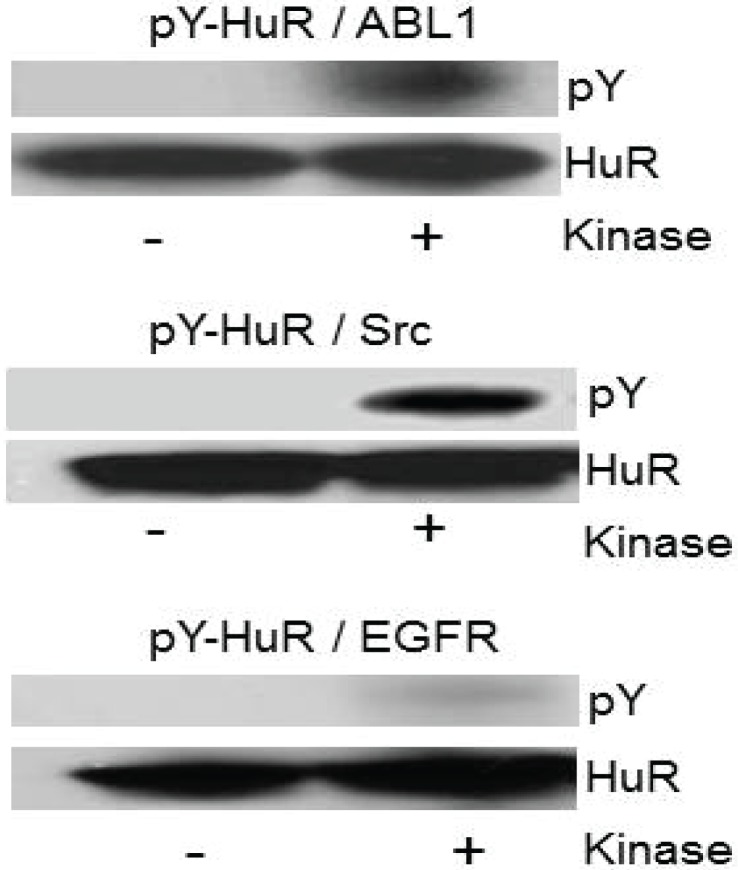
Recombinant HuR protein phosphorylation by Abl-1 and SRC kinases, *in vitro*. The phosphorylation of recombinant HuR protein (200 ng) by recombinant ABL1, Src and EGFR kinases detected with pY antibody in Western blots. Note the significant HuR phosphorylation by Abl-1 and SRC kinases.

In our next experiment, we evaluated the phosphorylation of recombinant HuR by recombinant SRC, Abl-1, and EGFR tyrosine kinases in a phosphorylation assay, *in vitro*. Figure 5 illustrates an example of HuR phosphorylation by the above-mentioned kinases detected by a pan phosphotyrosine antibody (pY). Note the significant phosphorylation of HuR protein detected by p-Y antibody following phosphorylation by Abl-1 and SRC kinases. Similar results have been observed in three experiments.

**Figure 6:**
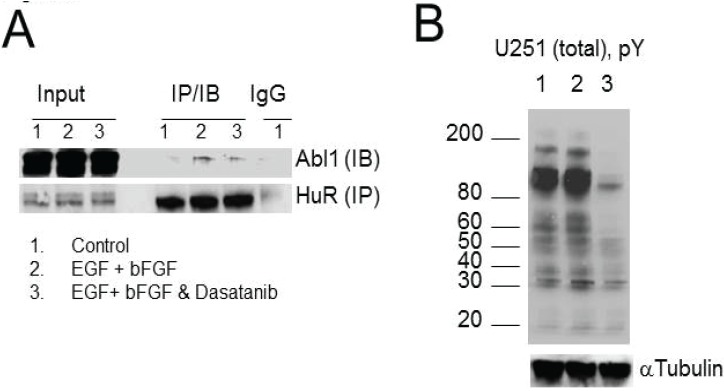
The interaction of HuR and Abl-1 kinase is growth factor dependent. (**A**) Immunoblotting for Abl-1 kinase co-immunoprecipitated with HuR from cells in the control condition, after treatment with growth factors, or growth factors in the presence of dasatinib. Note that the HuR and Abl-1 interaction is growth factor dependent and is diminished by dasatinib. The α-tubulin bands verify the equal gels loading. (**B**) The alteration of total pY phosphorylation detected with pY antibody in U251 cells treated with growth factors alone or in the presence of dasatinib, 500 nM compared to the control condition. The α-tubulin bands verify equal loading.

### 2.6. HuR Interaction with Abl-1 Kinase Is Growth Factor Dependent

To confirm that the growth factor dependent interaction of HuR with Abl-1 kinase occurs on the cellular level, we immunoprecipitated HuR and immunoblotted for Abl-1 kinase from cell lysates obtained in three conditions: 1—control; 2—following cell treatment with EGF and bFGF; and 3—following cell treatment with EGF and bFGF in the presence of dasatinib, 500 nM (the inhibitor of Abl and SRC tyrosine kinases families). We observed a significant enhancement of HuR association with Abl-1 kinase following treatment with growth factors, which was diminished by dasatinib (Figure 6A). Similar results have been achieved in three experiments. Figure 6B illustrates overall phosphorylation of tyrosine residues detected by p-Y antibody in lysates of U251 cells in control (first line), after treatment with EGF and bFGF (second line), and after treatment with EGF and bFGF in the presence of dasatinib (third line). Note the significant decline in overall pY signal following cell treatment with dasatinib.

This result confirms growth factor dependent interaction of Abl-1 kinase with HuR and resultant HuR phosphorylation by Abl-1.

### 2.7. The Consequences of Aberrant Growth Factor Dependent HuR Phosphorylation at Tyrosine Residues

**Figure 7:**
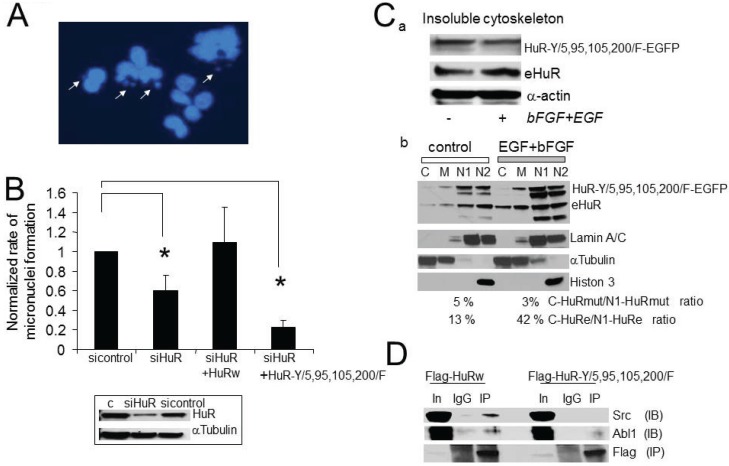
Increase in genomic instability is the consequence of growth factor dependent HuR phosphoregulation. (**A**) Illustration of micronuclei formation in U251 cells treated with growth factors. The micronuclei were detected with DAPI as isolated small DNA foci (marked with arrows). (**B**) The graph represents the averaged percentages of cells exhibiting micronuclei formation normalized to the value of micronuclei formation in sicontrol cells. The following conditions are shown: sicontrol, siHuR, siHuR plus HuRwild-EGFP, and siHuR plus HuR-Y/5, 95, 105, 200/F-EGFP. Note there is a significant decline in the percentages of cells with micronuclei in siHuR (0.60 ± 0.16, *n* = 3, *p* = 0.055) and siHuR plus HuR-Y/5,95,105,200/F-EGFP (0.23 ± 0.07, *n* = 3, *p* = 0.001) conditions compared to the sicontrol. All experiments were performed equally in the presence of growth factors (EGF, 40 nM and bFGF 40 nM, 48 h). The insert illustrates the decrease of endogenous HuR expression following transfection with siHuR RNA against 3'-UTR of HuR mRNA detected in Western blot. (**C**) The illustration of native HuR and HuR-Y/5,95,105,200/F-EGFP mutant proteins in insoluble cytoskeleton fractions before and after stimulation with growth factors detected in Western blot (a) and in soluble cytoplasmic, membrane, soluble nuclear, insoluble nuclear fractions (b). The fractions are marked as C-cytoplasmic, M-membrane, N1-soluble nuclear, N2-insoluble nuclear. (**D**) Illustration of co-immuno-precipitation of Src and Abl1 kinases with Flag-HuR and Flag-HuRmut proteins. The Flag-HuR and Flag-HuRmut proteins were immuno-precipitated by using Flag antibodies. The lines are marked as In—input (4%), IgG-immuno-precipitation with mouse IgG antibody, IP—immuno-precipitation with Flag antibody. The experiment has been performed in the presence of growth factors.

Abnormal centrosome amplification may lead to genomic instability during mitosis [5,6,7,8,9]. We predict that the growth factor dependent centrosome amplification in U251 cells may be accompanied by an increased rate of genomic instability which should be sensitive to alterations in HuR expression, sub-cellular distribution, or disruption in HuR phosphorylation at consensus sites for Abl-1 and Src kinases. The consensus sites (Y5, Y95, Y105, Y200) for phosphorylation by Abl-1 and SRC kinases are located in non-mRNA binding domains of HuR. To test our prediction, we compared micronuclei formation in U251-Teton cells in the control condition, after partial HuR knockdown, after the partial substitution of endogeneouse HuR to recombinant HuRwild-EGFP, or to recombinant HuR-Y/5,95,105,200/F-EGFP. All experiments were done in the presence of growth factors and at equal concentration of dox (0.35 μg/mL) for construct induction. The following four conditions have been used: 1—control (sicontrol RNA); 2—siHuR RNA (siHuR RNA against 3' UTR of endogenous HuR); 3—co-transfection with siHuR RNA and recombinant HuRwild-EGFP wild type construct (lacks the HuR 3' UTR so not silenced); and 4—co-transfection with siHuR RNA and recombinant HuR-Y/5,95,105,200/F-EGFP mutant construct (lacks the HuR 3' UTR so not silenced). In the control condition, the rates of genomic instability calculated as percentage of cells exhibiting micronuclei formation varied from 10% to 18%. Figure 7 represents the average rates of genomic instability normalized to the value of the rate of genomic instability in the control condition (sicontrol) for each experiment. At least 300 cells per condition have been analyzed in each experiment. We observed a significant decline in the rates of micronuclei formation following a decrease in HuR expression and after partial substitution of endogeneouse HuR to HuR-Y/5,95,105,200/F-EGFP mutant (0.60 ± 0.16 (*n* = 3) and 0.23 ± 0.07 (*n* = 3) are the normalized rates of genomic instability for siHuR and siHuR + HuR-Y/5,95,105,200/F mutant conditions, respectively). The substitution of endogeneouse HuR to HuRwild-EGFP did not significantly alter the rate of micronuclei formation (1.09 ± 0.37, *n* = 3). Note that the siHuR RNA against the 3' UTR of HuR has been used in the current experiments to diminish endogenous HuR protein expression by 40%–60% compared to si-control condition (Figure 7B, insert).

To verify the role of Y5,95,105,200 residues in HuR sub-cellular trafficking, we developed cell lines which stably express HuR-Y/5,95,105,200/F-GFP mutant and endogenous HuR, and compared sub-cellular distribution of above proteins in sub-cellular fractions after cell treatment with growth factors *versus* control. We confirmed a loss of growth factor dependent regulation of HuR-Y/5,95,105,200/F-GFP mutant in the insoluble cytoskeleton fraction and an abundance of the HuR-Y/5,95,105,200/F-EGFP mutant in the nuclear fractions (Figure 7C) compared to endogenous HuR. The value of endogenous HuR increased by 20%–80% in the insoluble cytoskeleton fraction following stimulation with growth factors; however, the value of HuR-Y/5,95,105,200/F-EGFP mutant analyzed in the same insoluble cytoskeleton fractions stayed the same or declined by 0%–20% after stimulation with growth factors (Figure 7Ca). The ratio of cytoplasmic soluble to nuclear soluble was 46% ± 8% (*n* = 3) for endogenous HuR compared to 10% ± 8% (*n* = 3) for HuR-Y/5,95,105,200/F-EGFP in the presence of growth factors (Figure 7Cb). The difference is significant (*p* = 0.05, *n* = 3).

Co-immunoprecipitation of SRC and Abl-1 kinases with recombinant Flag-HuRwild and Flag-HuR-Y/5,95,105,200/F mutant (IP with Flag antibody) confirms a decline in the interaction of SRC and Abl-1 kinases with the Flag-HuRmut protein compared Flag-HuRwild (Figure 7D). A mouse IgG antibody was used for control immunoprecipitation. Similar results have been observed in two experiments.

These results emphasize the essential role of HuR tyrosine residues (Y/5, 95, 105, 200/) in growth factor dependent HuR regulation promoting genomic instability in U251 cells.

### 2.8. HuR, Abl-1 and SRC Expression in Tumor Samples and Glioma Cell Lines

**Figure 8:**
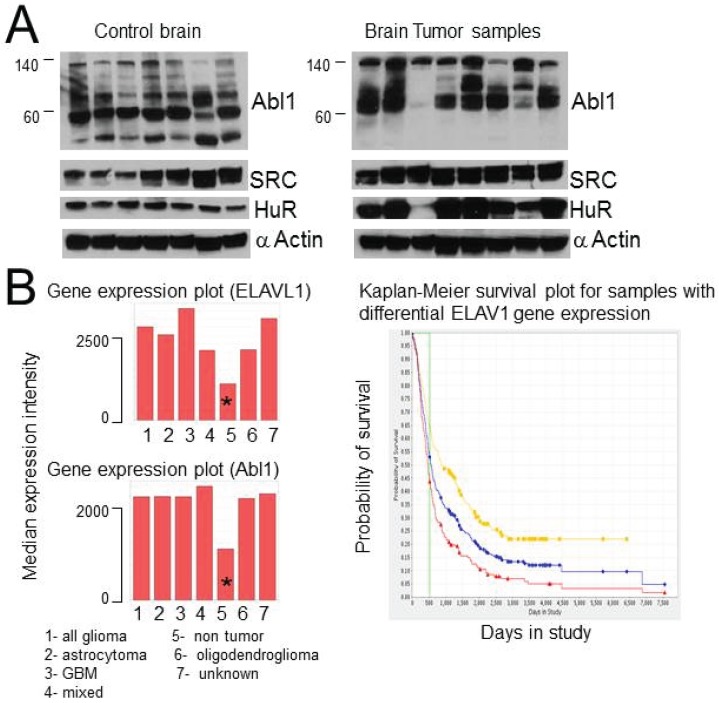
The expression of HuR, SRC, and Abl-1 in brain tumor and control samples. (**A**) Illustration of expression of HuR, SRC, and Abl-1 proteins in samples from brain tumor and control brain tissue. (**B**) Left—Profiles of HuR and Abl-1 mRNAs expression in different groups of brain tumors compared to non-tumor group. The data was obtained from Rembrandt National Cancer Institute database. * represents non-tumor control brain. Right—Illustrates Kaplan-Meier survival plot for GBM patients with different levels of ELAVL1 gene expression (201727—Taqman probe for ELAVL1 gene detection). The survival curves represent the following groups: red—up regulated (192), yellow—intermediate (150), green—down regulated (1), blue—all (343). Log-rank p-value for significance of different survival between up-regulated *versus* intermediate groups is 1.929E-7 (Rembrandt National Cancer Institute data base).

To verify that the expression and possible interaction of HuR with Abl-1 and SRC kinases is not restricted to U251 cells, we analyzed Abl-1, SRC, and HuR expression in eight brain tumor samples and seven samples of control brain tissue (Figure 8A). We also checked five additional established glioma cell lines. We confirm over-expression of HuR and Abl-1 in the majority of tumor samples and cell lines compared to control brain tissues (Figure 8A). In addition, we noticed the unique pattern of Abl-1 expression (cleavage or isoforms) in tumor samples, which was different from the Abl-1 pattern in samples of control brain tissues (Figure 8A). The HuR and Abl-1 protein expression data is in agreement with the profiles of mRNA expression of HuR (ELAVL1) and Abl-1 genes provided in the Rembrandt National Cancer Institute database (Figure 8B). Figure 8B illustrates a significant increase (over two fold) in HuR and Abl-1 mRNA expression in groups of different types of brain tumors compared to the normal (non tumor) group. In addition, over-expression of HuR is associated with decreased patient survival as shown in the Kaplan-Meir survival curves (Figure 8B-right).

HuR is a mRNA binding protein involved in tumor cell survival, proliferation and migration [17,18,19,20,37]. The main HuR function is the stabilization of mRNA transcripts through the binding to the U (uridine) rich motifs in 3' UTR (untranslated mRNA region) [24,38]. In our current report, we reveal a new HuR role in genomic instability through a growth factor dependent control of centrosome function.

Mitotic errors are common in cancer and occur at a high rate in GBM [34,35]. Abnormalities in centrosomal/chromosomal networks and in chromosomal recombinations are underlying mechanisms of genomic instability in cancer cells [6,7]. The integrity and function of the centrosomal/chromosomal network in mitotic cells is coordinated by growth factor and extracellular matrix dependent signaling pathways [8,10,11,12,13,14,15,39], which have a high degree of diversity in the brain and are abnormal in more than 50% of primary brain tumors [40,41,42,43]. Indeed, the brain microenvironment consisting of a high concentration and variety of growth factors may explain the high frequency of genomic alterations events observed in GBM compared to other types of cancer. It is well documented that hyperactivity in EGF, bFGF, and PDGF dependent pathways is a leading contributor to centrosome amplification in tumors with aberrant check point mechanisms [5,8,12,14,40]. In agreement with previous observations, we demonstrated the EGF and bFGF dependent amplification of centrosome number in U251 cells and revealed a novel post-transcriptional regulator of this process.

Several families of tyrosine kinases (SRC, EGFR, FGFR, Abl-1) have been reported to be directly involved in growth factor dependent regulation of centrosome structure and function in cancer cells [8,31,32,33]. Growth factor dependent regulation of translation in centrosomes has been observed in anaplastic large cell lymphomas [26,27]. We found an association of SRC and Abl-1 tyrosine kinases with centrosomes in U251 cells. We consider Abl-1 and SRC kinases as the upstream regulators of growth factor dependent HuR association with the pericentriolar matrix. We confirmed a growth factor dependent interaction of HuR with Abl-1 and SRC kinases at the cellular level and phosphorylation of recombinant HuR by Abl-1 and SRC kinases, *in vitro*. Recently, we provided evidence of HuR dependent translation occurring in the pericentriolar matrix in U251 cells [16]. We linked cyclin A protein level in centrosomes directly to the HuR (protein)/cyclinA(mRNA) interaction which affected cyclin A mRNA stabilization and translation in centrosomes. We consider this type of centrosome content regulation by HuR as a direct HuR influence on centrosomes. We propose that growth factor dependent HuR association with centrosomes may enhance translation in the pericentriolar matrix and thus, may induce abnormal centrosome amplification.

The process of posttranslational HuR regulation by phosphorylation is implicated in the HuR sub-cellular localization, mRNA binding, and degradation [16,20,22,44]. The majority of these regulations rely on modification of serine and threonine residues in HuR [44]. The activation of EGF and bFGF signaling pathways in cancer cells leads to alteration of both tyrosine kinases and serine/threonine kinases [45], which may influence HuR function. We have described the regulation of centrosome function associated with posttranslational HuR modification by tyrosine kinases. We propose that HuR regulation by SRC and Abl-1 tyrosine kinases may facilitate the HuR intracellular transport and ability to be involved in translation among different sub-cellular compartments.

HuR participates in the regulation of hundreds of mRNA transcripts [21,24] including more than 70 genes that may regulate homo/hetero chromosomal recombinations and centrosome amplification, cohesion, and clustering. In our manuscript, we report that the one of main consequences of abnormal HuR dependent regulation of centrosomes is augmentation of genomic instability. The trio of centrosome aberration, increase of genomic instability, and the facilitation of cell survival provided by HuR may underlie a mechanism of tumor heterogeneity and progression. HuR expression has been assigned as a negative prognostic factor in several types of cancer [37,46,47]. In agreement with our observation, others have identified HuR as an oncogene [48,49], which may induce the DNA damage program when over expressed. On the other hand, HuR sub-cellular redistribution has been reported as a first response to different types of geno-toxic stresses [44,50,51,52] during the initiation of apoptosis. The divergence between apoptosis and genomic instability is a complex process that is regulated by several cellular mechanisms like check point molecules, cytoskeletal structures, and mitotic status [5,6,9,25]. The HuR dependent regulation of these processes may vary between different cancers or even within tumors leading to tumor progression or to mitotic catastrophe and cell death depending on the tumor genotype and microenvironment. Thus, HuR dependent centrosome regulation may be either cancerogenic or apoptotic depending on the tumor genotype, phenotype, and microenvironment.

In summary, we highlight a novel function for HuR in induction of growth factor dependent genomic instability in cancer cells and provide a possible mechanism for HuR dependent regulation of centrosome function.

## 3. Experimental Section

*DNA Constructs, Stable Cells Line Creation, Cell Culture Procedure*. PACT-mKO1 (PACT domain of pericentrin attached to the red fluorescence protein), HuR-EGFP (HuR attached to the green fluorescence protein), HuR-Y/5,95,105,200/F-EGFP, Flag-HuR, Flag-HuR-Y/5,95,105,200/F and HuR-pGEX-6P constructs were used in the current manuscript. The mutations of Y (tyrosine) at the 5, 95, 105, 200 residues of HuR to F (phenylalanine) were achieved by using QuikChangeTM site-directed mutagenesis kit (Stratagene) and the following primers: Y5F-forv-5'-GTCTAATGGTTTTGAAGACCACATG and Y5F-rev-5'-CATGTGGTCTTCAAAACCATTAGAC; Y95F-forv-5'-AAGGTGTCGTTTGCTCGCCCG and Y95F-rev-5'-CGGGCGAGCAAACGACACCTT; Y105F-forv-5'-GCCAACTTGTTCATCAGCGGG and Y105F-rev-5'-CCCGCTGATGAACAAGTTGGC; Y200F-forv-5'-CTCTCGCAGCTGTTCCACTCGCCAG and Y200F-rev-5'-CTGGCGAGTGGAACAGCTGCGAGAG. All constructs were confirmed by sequencing. The stable cell lines for conditioning construct expression were created by using the Tet-ON system (Clontech Laboratories, Inc., Mountain View, CA, USA) in U251 cells. Cells were expanded in DMEM-F12 medium supplemented with FBS (10%) or FBS (0%) for synchronization, L-glutamine and penicilin/streptomycine were added in the solution. The stable cell medium was supplemented with G418 and hydromycin B to ensure clone selection. The stimulation with growth factor was performed by using EGF (Sigma, St. Louis, MO, USA) and bFGF (Sigma) in serum free medium.

Tumor and control tissues were provided by the UAB IRB-approved Brain Tumor Tissue Core directed by Yancey Gillespie, PhD.

*Phosphorylation Assay*, *in Vitro*. The recombinant HuR protein phosphorylation by recombinant c-Abl, EGFR, c-SRC kinases (ProQinase GmbH, Freiburg, Germany) and (New England Biolabs Inc., Beverly, MA, USA) were performed at 30 °C for 40–60 min in Kinase buffer (Cell Signaling Technology, Danvers, MA, USA) supplemented with Mg-ATP. The recombinant HuR-GST protein was purified by using 5 mL GSTrap HP columns (GE Healthcare Bio-Sciences, Pittsburgh, PA, USA), dialyzed against PBS in 3–12 mL dialysis cassettes (Thermo Scientific, Rockford, IL, USA), the GST was truncated from HuR with thrombin [8].

*HuR Protein Knockdown*. For HuR protein knockdown, the ELAV1 sh-plasmids KH 13883N (Sillencing TM shRNA plasmid) and as a negative control the sh-control plasmid KH 13883N were used at equal concentrations for cell transfection (Lonza, Koln, Germany). For decrease of endogenous HuR protein expression in the HuR substitution experiments, the HuR siRNA 1027417 (Qiagen GmbH, Hilden, Germany) against 3' UTR of HuR mRNA was used for transfection and the scrambled siRNA (Cell signaling Technology) was used as a negative control. The HuR substitution experiments have been performed on U251 Teton cells equally treated with 0.35 μg/mL dox in all conditions. The following conditions have been used: 1—control (sicontrol RNA); 2—siHuR RNA (siHuR RNA against non cording 3' UTR of endogenous HuR); 3—siHuR RNA against 3' UTR co-transfected with dox inducible recombinant HuRwild-EGFP construct; and 4—siHuR RNA against 3' UTR co-transfected with dox inducible recombinant HuR-Y/5,95,105,200/F-EGFP construct. The protein contents were analyzed 48–72 h after transfection.

*Protein Co-Immunoprecipitation, Fractionation, and Western Blotting*. The immuno-precipitations were performed by using appropriate mouse/or rabbit IP matrixes (Santa Cruz Biotechnology, Inc.) coated with HuR (HuR3A2 antibody, Santa Cruz Biotechnology, Inc., Dallas, TEXAS, USA), or Flag (Sigma), or γ-tubulin (Santa Cruz Biotechnology, Inc. or Cell Signaling Technology) antibodies, or anti-mouse/ or anti-rabbit IgG antibodies as control (Santa Cruz Biotechnology, Inc.) overnight at 4 °C. The cells lysates were pre-cleaned for 2 h with immobilized A/G protein slurry (Life Technologies, Grand Island, NY, USA) before immunoprecipitation. The immunoprecipitation was achieved during period of 6 h at 4 °C, the beads were washed 3–5 times in appropriate buffers at 4 °C and contents were released by using 5× SDS gel loading buffer and 95 °C heat for 8 min. The cell lysis buffer (Cell Signaling Technology, Boston, MA, USA) supplemented with protease inhibitor cocktail (Thermo scientific) and sodium orthovanadate was utilized for cells lysis. The 4%–5% blotting grade blocker (Bio-Rad) or 3.5% BSA (during pY detection) in TBST have been used as blocking solutions for Western blots. The protein fractionations have been performed by using nuclear/cytoplasmic kit (Pierce Biotechnology, Rockford, IL, USA) or subcellular protein fractionation kit for cultured cells (Thermo Scientific) with according protocols. The α-tubulin, lamin A/C, Histon3 antibodies were used as markers for cytoplasmic, nuclear and nuclear insoluble fractions, respectively. The following antibodies were used in immunoblottings: p-c-Abl (Y412), c-Abl, p-Sr family (Y416), c-SRC, Lyn, EGFR, Lamin A/C, p-Tyrosine-1000, γ-Tubulin, GAPDH (Cell Signaling Technology, Boston, MA, USA), purified mouse anti-phosphotyrosine (BD Biosciences, San Jose, CA, USA), HuR3A2, HuR-H280, actin, γ-Tubulin (Santa Cruz Biotechnology), anti-Cdk5Rap2 (EMD Millipore, Billerica, MA, USA), α-Tubulin, Flag (Sigma), Histon3 (Calbiochem International, La Jolla, CA, USA).

*Centrosome purification and fractionation* have been performed as previously described [16]. Briefly, in the first step the cytoplasmic fraction was separated from the nuclear fraction by centrifugation at 15,000× *g* for 5 min at 4 °C after cells exposure to solutions with different osmolarity (PBS, 0.1 PBS, 8% sucrose in 0.1× PBS, 8% sucrose in H2O, 1 mM Tris-Cl for 2 s at 4 °C) and cells lysis; in the second step the crude centrosomes fraction was isolated by centrifugation in Ficoll cushion solution at 25,000× *g* for 20 min at 4 °C; in the third step the pure centrosomes fraction was obtained by centrifugation in a discontinuous 62.5%–20% sucrose density gradient solution at 96,500× *g* for 2 h at 4 °C and fractionation. The α-tubulin, lamin A/C, and γ-tubulin were used as markers for cytoplasmic, nuclear and centrosome fractions, respectively.

*Fluorescence microscopy* was performed using an Olympus DP71 fluorescence microscope with DP controller 3.3.1.294 system (Olympus, Center Valley, PA, USA) and Nikon Eclipse Ti fluorescence microscope with NIS-Elements software (Nikon Inc., Melville, NY, USA). The fluorescence signals from HuR-EGFP, PACT-mKO1 constructs and from nuclei marked with DAPI were detected from both, living cells and fixed cells by using 40× and 60× objectives. The cells fixation was done in 3.7% solution of paraformaldehyde for 13 min at room temperature.

*Rembrandt Database Analysis*. The National Cancer Institute REMBRANDT database has been accessed through the https://caintegrator.nci.nih.gov/rembrandt/ website. The ELAVL1 gene expression plot is illustrated for probesets 1994. The Abl1 gene expression plot is illustrated for probesets 202123_s_at. The abbreviations of group names are: 1—all glioma (454), 2—astrocytoma (148), 3—GBM (228), 4—mixed (11), 5—non-tumor (28), 6—oligodendroglioma (67), and 7—unknown (67). The Kaplan-Meier survival plot for samples with differential ELAVL1 gene expression is illustrated for Affymetrix reporter 201727_s_at (prob hit starts at position 733 and hit ends at position 1240 of the stretch of mRNA of ELAVL1 gene, NM001419.2. Target transcript is 6058 bp long, cording sequence starts at position 168, ends at position 1148, and the 3' UTR is 4909 bp long.

*Statistical analysis and graphing* were performed using Excel and OriginPro software. The statistical significance was determined by Student’s t-test and was considered significant at the *p* ≤ 0.05. The statistically significant data are labeled by an asterisk in the graphs.

## 4. Conclusions

In summary, our paper highlights a new node regulating centrosome amplification and genomic instability in cancer cells. We report on a growth factor dependent mechanism of centrosome regulation by the mRNA binding protein HuR. We found that the co-localization of HuR with the pericentriolar matrix (PCM) is a growth factor dependent process and facilitates centrosome amplification. SRC and Abl-1 kinases direct the growth factor dependent HuR sub-cellular trafficking and association with the PCM. SRC and Abl-1 kinases interact with HuR, *in vitro*, and phosphorylate recombinant HuR. HuR appears to be involved in the regulation of genomic instability through a growth factor mediated and centrosome dependent pathway in glioma.
